# A long-term monitoring dataset of non-breeding waterbirds at Lake Miedwie, Poland (2002–2025)

**DOI:** 10.3897/BDJ.13.e160615

**Published:** 2025-08-04

**Authors:** Dominik Marchowski, Paweł Stańczak, Michał Jasiński, Sebastian Guentzel

**Affiliations:** 1 Ornithological Station, Museum and Institute of Zoology, Polish Academy of Sciences, Gdansk, Poland Ornithological Station, Museum and Institute of Zoology, Polish Academy of Sciences Gdansk Poland; 2 West Pomeranian Nature Society, Szczecin, Poland West Pomeranian Nature Society Szczecin Poland

**Keywords:** TRIM software, International Waterbird Census, Natura 2000, Important Bird and Biodiversity Area, abundance trends, standardised bird counts, wetland ecosystem, Anseralbifrons, Aythyaferina, Anasplatyrhynchos

## Abstract

**Background:**

Lake Miedwie, located in north-western Poland, is one of the most important wetland areas for non-breeding waterbirds in the Central European region. Recognised as both an Important Bird Area (IBA) and part of the Natura 2000 network, the site supports large numbers of migratory and overwintering geese, ducks and other waterbirds. Since 2002, standardised surveys have been conducted during the non-breeding season to monitor population sizes and species composition. The site’s location on major flyways and its habitat diversity make it valuable for long-term ornithological monitoring.

**New information:**

This dataset presents waterbird count data collected during the non-breeding season from 2002/2003 to 2024/2025, covering 23 seasons across 24 calendar years. The dataset includes 952 records of seasonal abundance for 14 waterbird species. The average total abundance was 7,933 (± 1,314 SE) individuals per season (mean of November, January and March), with a maximum of 36,095 in November 2002 and a minimum of 226 in March 2017. Trend analysis using the rtrim package in R indicated a moderate overall decline (λ = 0.976, 95% CI: [0.975 – 0.977]). Species-specific trends varied, with some taxa, such as Greylag Goose (*Anseranser* Linnaeus, 1758), increasing, while others, including Greater White-fronted Goose (*Anseralbifrons* Scopoli, 1769), Eurasian Teal (*Anascrecca* Linnaeus, 1758) and Eurasian Coot (*Fulicaatra* Linnaeus, 1758), declined. The dataset contributes valuable baseline data for wetland bird conservation, ecological assessment and future research in Central Europe. Importantly, such long-term monitoring also provides a robust reference point for assessing changes in climate and biodiversity over time.

## Introduction

Lake Miedwie is one of the largest and most ecologically important inland lakes in Poland. It is located in the Pomeranian Lakeland and forms the core of a larger wetland complex that includes the Płoń River Valley, smaller glacial lakes and extensive marshes. This mosaic of habitats supports rich aquatic and wetland bird communities and is recognised as both an Important Bird and Biodiversity Area (IBA) (Kalisiński et al. 2010) and a Natura 2000 Special Protection Area (SPA) under the EU Birds Directive (Polish name: Jezioro Miedwie i okolice, English name: Miedwie Site, area code: PLB320005).

The importance of Lake Miedwie for waterbirds is rooted in its strategic location along migratory flyways and its high availability of food and roosting habitats during the non-breeding season. Since 2002, the area has been systematically monitored as part of the International Waterbird Census (IWC), coordinated by Wetlands International (Wetlands International 2010). Monitoring focuses on the non-breeding period, typically covering counts in November, January and March, corresponding to autumn, mid-winter and early spring migration periods.

Long-term monitoring of bird populations is crucial for detecting temporal trends, understanding shifts in community composition and providing information for conservation priorities (Wetlands International 2010, Chylerecki et al. 2015, Marchowski et al. 2017, Marchowski et al. 2018). This information is particularly relevant in wetlands that support large aggregations of waterbirds and are exposed to various pressures, including climate change (Marchowski et al. 2017), habitat alteration (Marchowski et al. 2020) and disturbance (Sikora et al. 2011).

This data paper documents a 23-seasons' dataset of waterbird counts from Lake Miedwie and provides descriptive analyses of population trends and species composition. In addition to presenting data structure and methodology, we summarise average abundances and inter-annual variation for the most species. The aim is to provide a comprehensive reference point for conservationists, policy-makers and researchers working on waterbird ecology and wetland management.

## General description

### Purpose

This dataset provides long-term, standardised information on the seasonal abundance and species composition of non-breeding waterbirds at Lake Miedwie, Poland. By making these data publicly accessible, the dataset supports future ecological assessments, conservation planning and trend analyses. It also contributes to improving the availability of high-quality monitoring data from Central Europe, promoting reuse in studies on wetland biodiversity and environmental change.

This dataset follows FAIR principles by being findable via DOI, openly accessible through the GBIF data portal and structured according to the Darwin Core standard as a sampling-event dataset. It includes well-documented metadata and standardised fields that ensure interoperability with biodiversity data systems and support long-term reuse. The dataset contributes to the evidence base for understanding waterbird population dynamics and changes in wetland ecosystems.

### Additional information

The dataset comprises 952 records of non-breeding season waterbird counts collected across 23 monitoring seasons (2002/2003–2024/2025) at Lake Miedwie. A full season was defined as the set of results from three coordinated waterbird counts conducted within a single non-breeding season: in November, January and March (e.g. November 2002, January 2003 and March 2003 for the 2002/2003 season). The most recent season (2024/2025) is incomplete, as it includes counts from November 2024 and January 2025, but lacks data for March 2025. Additional data gaps include missing counts for March and January 2005, as well as for November, January and March 2008.

**Figure 1.**
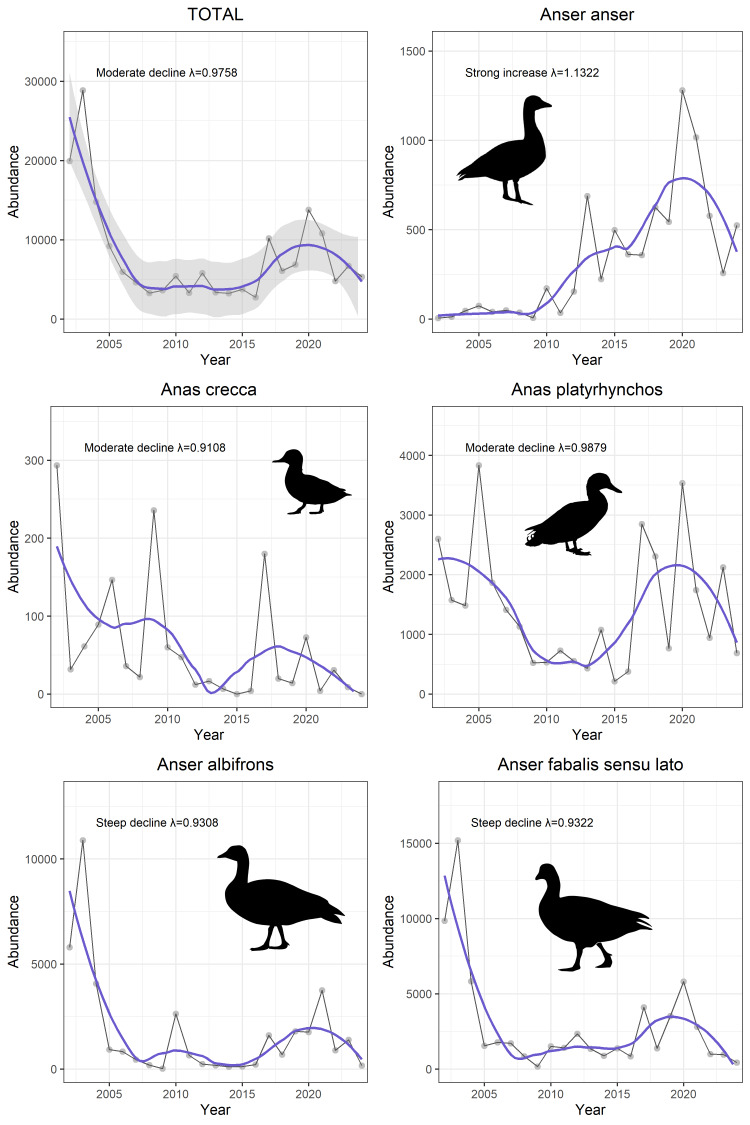
Long-term population trends of selected waterbird species at Lake Miedwie during the non-breeding seasons 2002/2003–2024/2025. The blue line represents a smoothed trend with 95% confidence intervals (grey shading in TOTAL only). Total waterbird abundance and six representative species are shown, including species with contrasting patterns (e.g. *Anseranser* – strong increase; *Anascrecca*, *Anseralbifrons* – moderate to steep declines). Trend estimates (λ) were calculated using TRIM software, based on standardised count data.

These records cover 14 key waterbird species and one aggregate category representing total seasonal abundance. The average abundance of all analysed species across the study period was 7,933 individuals per season (± 1,314 SE). Substantial interannual variation was observed. The highest total occurred in November 2002 with 36,095 individuals, while the lowest was recorded in March 2017 with 226 individuals. Long-term trend analysis using the rtrim package in R indicated a moderate population decline, with an estimated average annual growth rate of λ = 0.976 (95%CI [0.975 – 0.977]). The decline was most pronounced between the 2003 and 2010 seasons, followed by a period of stabilisation and slight recovery. Despite this, total abundances remained consistently below the initial baseline in subsequent years (Fig. 1).

To illustrate changes in waterbird abundance over time, we compared the first six full seasons (2002/2003–2007/2008) with the last six (2018/2019–2023/2024). These periods were selected to represent the beginning and end of the monitoring programme, while averaging over six seasons helped to minimise the influence of short-term fluctuations and anomalous years. This approach provides a more robust snapshot of typical abundance during each period, without being overly affected by either single-season extremes or long-term population trends (Table 1).

TRIM trend analysis was performed for the period 2002/2003–2024/2025 (23 seasons), including the season 2024/2025, which was only partially completed at the time of analysis. This was possible because the TRIM software, based on Generalised Estimating Equations (GEE), can handle missing values in the dataset and provides robust estimates even in the presence of incomplete data (Pannekoek and van Strien 2005). In contrast, the population size comparison between the First6 and Last6 periods was based exclusively on fully completed seasons (up to 2023/2024), as these raw averages were calculated directly without any model-based imputation. To ensure comparability, only seasons of equal data quality and completeness were used in this comparison.

## Project description

### Title

Monitoring Wintering Waterbirds in West Pomerania

### Personnel

The fieldwork was carried out by professional ornithologists with biological education, affiliated with the West Pomeranian Nature Society (Zachodniopomorskie Towarzystwo Przyrodnicze, Szczecin, Poland).

### Study area description

The study was conducted at Lake Miedwie and adjacent wetlands in north-western Poland, within the boundaries of a designated Important Bird and Biodiversity Area (IBA). This region comprises one of the largest lowland lakes in Poland, surrounded by wet meadows, oxbow lakes and rivers. The area supports significant concentrations of waterbirds during the non-breeding season and has been recognised as a Natura 2000 site due to its ecological value (Polish name: Jezioro Miedwie i okolice, English name: Miedwie Site, area code: PLB320005).

### Design description

The project is part of a long-term monitoring scheme aimed at assessing changes in the abundance and seasonal dynamics of key waterbird species. Standardised shore-based surveys were conducted three times per season (November, January and March) from 2002/2003 to 2024/2025. The methodology followed protocols developed by Wetlands International under the International Waterbird Census (IWC, Wetlands International (2010)), ensuring consistency and comparability of data over time.

### Funding

The monitoring was conducted as part of a programme coordinated by the West Pomeranian Nature Society (Zachodniopomorskie Towarzystwo Przyrodnicze, Szczecin, Poland). Additional data were collected within the framework of the Polish Waterbird Monitoring Scheme, coordinated by the consortium of the Museum and Institute of Zoology, Polish Academy of Sciences (Muzeum i Instytut Zoologii PAN) and the Polish Society for the Protection of Birds (Ogólnopolskie Towarzystwo Ochrony Ptaków, OTOP), under commission from the Chief Inspectorate of Environmental Protection (Główny Inspektorat Ochrony Środowiska). Since this is a long-term programme with periodically renewed contracts, we refer interested readers to the official website for details on the project: https://monitoringptakow.gios.gov.pl/about-project.html.

## Sampling methods

### Study extent

The study was conducted at Lake Miedwie and adjacent wetlands in north-western Poland, a region designated as an Important Bird and Biodiversity Area (IBA). The monitoring covered the non-breeding season across three periods annually: November (autumn migration), January (mid-winter) and March (early spring migration). Observations were performed between the 2002/2003 and 2024/2025 seasons, resulting in a 23-seasons' continuous dataset.

### Sampling description

Waterbird counts were carried out using shore-based surveys with binoculars and spotting scopes. Observations were performed either by walking along predefined shoreline routes or from fixed vantage points, ensuring complete spatial coverage of the site. Binoculars with 10× magnification were used, while spotting scopes were typically equipped with zoom lenses ranging from 30× to 60×, occasionally up to 70×. The monitoring followed the international standards of Wetlands International and the methodology of the International Waterbird Census (Wetlands International 2010).

### Quality control

All observers involved in the monitoring were experienced field ornithologists with formal biological training. Data collection was standardised across years and periodic consultations were held amongst team members to ensure consistency in field methods and identification practices. Anomalous values were verified against original field notes. Zero counts were recorded explicitly in the digitised dataset and gaps in data collection were documented within the event file following the Darwin Core standard.

### Step description

To assess long-term trends, we calculated the average population growth rate (λ) for each species using the rtrim (Trends and Indices for Monitoring data) package in the R environment (R Core Team 2025), developed by Statistics Netherlands (Pannekoek and van Strien 2005). Rtrim applies Generalised Estimating Equation (GEE) models with Poisson error distributions, which account for serial autocorrelation and allow for estimation of missing values. Yearly indices of abundance are expressed relative to the first monitoring season (2002/2003). In this framework, the expected abundance *μ_it_* for site *i* and year *t* is modelled using the equation:

\begin{varwidth}{50in}\begin{equation*}
            log⁡(\mu_it) =\alpha_i+\beta_t
        \end{equation*}\end{varwidth}

where *α_i_* represents the site effect and *β_t_* the effect of year *t.* The overall population growth rate λ is then derived as:

\begin{varwidth}{50in}\begin{equation*}
            \lambda= e^\beta ® 
        \end{equation*}\end{varwidth}

where *β* denotes the average yearly change in abundance on a log scale. This allows estimation of mean annual change in population size (Pannekoek and van Strien 2005).

The calculated λ values represent average population changes over time, while confidence intervals provide information on uncertainty. Trend classification (e.g. steep decline, moderate decline, stable, uncertain, moderate increase and strong increase) follows standard TRIM categories and is based on the width of the confidence intervals (Pannekoek and van Strien 2005). Higher estimation error reduces the likelihood of identifying statistically significant trends, potentially masking actual changes in population size (Wetlands International 2010).

Early–Late Mean Comparison (ELMC)

In addition to the TRIM-based trend analysis, we applied a complementary approach (ELMC) to quantify changes in species abundance by comparing the early and late phases of the monitoring period. This method does not aim to model long-term trends, but rather highlights the magnitude of change between two time windows, which can be more illustrative for some species, such as geese, that show irregular or non-linear dynamics. To estimate average seasonal abundance per species, we first calculated the mean of the three monthly counts conducted within each monitoring season (November, January and March; e.g. November 2010, January 2011 and March 2011 formed the 2010/2011 season). These seasonal means were then averaged across the first six and the last six monitoring seasons to assess overall change in abundance.

The use of six-season windows at the beginning and end of the monitoring period provides a robust basis for comparison by minimising the influence of short-term fluctuations and outlier years, while avoiding dilution of the signal by long-term trends. This approach is particularly useful for species with irregular or episodic abundance patterns, where modelling continuous trends may be less informative. It complements the TRIM-based analysis by offering a more direct, model-free perspective on the magnitude of change over time.

## Geographic coverage

### Description

The Miedwie Site, a designated Important Bird and Biodiversity Area (IBA) of international significance (Kalisiński et al. 2010, Fig. 2), was chosen for this case study. It is located in north-western Poland, within the West Pomeranian Voivodeship and overlaps with the Natura 2000 Special Protection Area “Jezioro Miedwie i okolice” (site code: PLB320005), which was designated under the EU Birds Directive in 2004 and covers 16,510.98 ha (165.11 km²) (European Commission 2024).

The monitoring data presented here, however, do not cover the entire IBA or Natura 2000 area. Instead, they originate specifically from the main lake body — Lake Miedwie — and the adjacent wetland habitats forming a coherent natural unit along its shoreline.

Lake Miedwie is the fifth-largest lake in Poland and the second-largest in the West Pomeranian Region, with a surface area of 3,527 ha (35.27 km²) (Miedwie 2022). It is situated at an elevation of 14 m a.s.l. and reaches a maximum depth of 43.8 m, making it the country’s largest cryptodepression. The shoreline extends approximately 39 km, with a maximum width of 3.2 km. The shores are mostly treeless, with marshy zones particularly in the southern part. The Lake is fed by several tributaries, including the Ostrowica, Miedwinka and Gowienica Miedwiańska rivers, while the Płonia River flows through it. Near the Village of Żelewo, the Lake also serves as a source of drinking water for the City of Szczecin (Jańczak et al. 1996, Kalisiński et al. 2010).

The approximate central coordinates of the Miedwie Site are 53.2649° N, 14.8819° E.

## Traits coverage

This dataset includes seasonal counts of 14 waterbird species occurring during the non-breeding season at Lake Miedwie. The selection comprises both widespread and conservation-relevant taxa representing Anatidae and Rallidae families, along with several species of geese and diving ducks. These species were chosen, based on their regular occurrence in the study area and their ecological relevance to wetland monitoring (Kalisiński et al. 2010). An aggregated "TOTAL" category was also used to represent the overall abundance of all waterbirds recorded in each season.

While 14 species were selected for analysis, this subset does not represent the full spectrum of waterbirds at Lake Miedwie. For instance, swans (*Cygnus* spp.) and the Great Crested Grebe (*Podicepscristatus*) were excluded. The selection was guided by species’ representativeness for the area and their susceptibility to hunting pressure (Marchowski et al. 2025b).

Table 2 provides the list of species included in the dataset with full scientific names and authorities.

## Temporal coverage

### Notes

**Figure 3.**
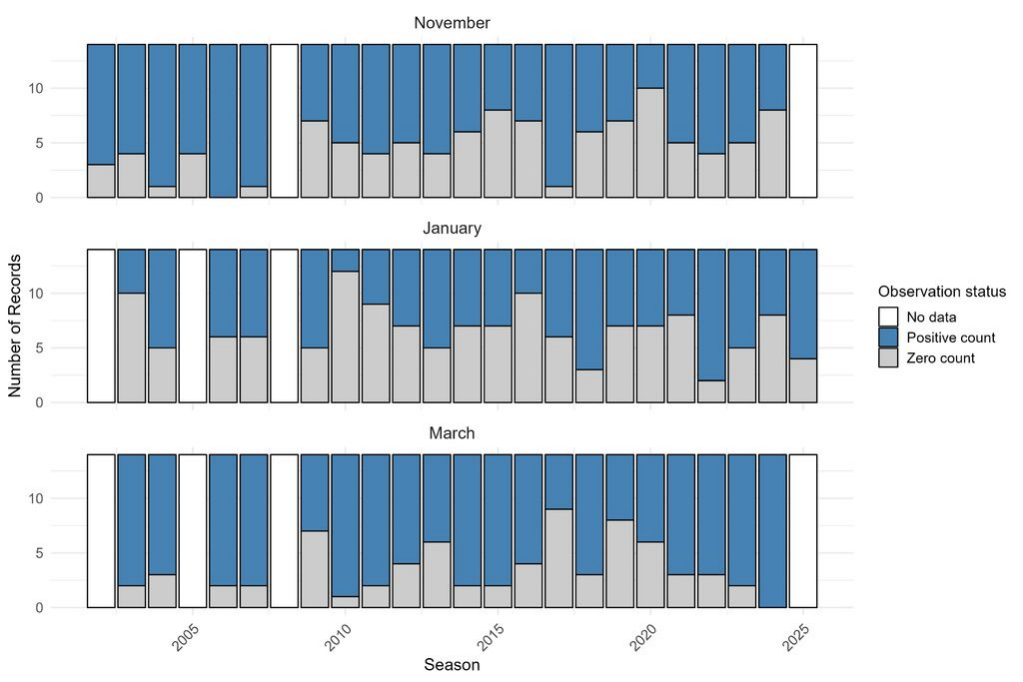
Number of waterbird species records collected at Lake Miedwie during the non-breeding season (November, January, March) in each monitoring season from 2002/2003 to 2024/2025, categorised by observation type: positive counts, zero counts and missing data. Gaps indicate months where no field observations were recorded.

This dataset spans 23 non-breeding seasons, from 2002/2003 to 2024/2025. Waterbird surveys were conducted three times annually (in November, January and March), capturing both overwintering and early-migratory individuals. A total of 952 species-season-month combinations were recorded. The number of observations per season varied slightly, but remained consistent over the years, providing a robust basis for long-term analysis of bird population trends and interannual variation (Fig. 3).

## Usage licence

### Usage licence

Other

### IP rights notes

This dataset is published under the Creative Commons Attribution 4.0 International (CC BY 4.0) licence. Users are free to share and adapt the material for any purpose, provided appropriate credit is given to the original authors and source.

## Data resources

### Data package title

A long-term monitoring dataset of waterbirds at Lake Miedwie, Poland (2002–2024). Museum and Institute of Zoology, Polish Academy of Sciences. Sampling event dataset.

### Resource link

https://doi.org/10.15468/xcvaee

### Alternative identifiers

https://ipt.gbif.pl/resource?r=miiz-miedwie

### Number of data sets

1

### Data set 1.

#### Data set name

A long-term monitoring dataset of waterbirds at Lake Miedwie, Poland (2002–2024).

#### Data format

Darwin Core Archive (DwC-A) - Sampling Event dataset.

#### Character set

UTF-8

#### Download URL

https://ipt.gbif.pl/archive.do?r=miiz-miedwie&v=1.1

#### Data format version

1

#### Description

This dataset presents long-term shore-based monitoring of waterbirds at Lake Miedwie, Poland, from 2002 to 2024. Monitoring based on and extending the methodology of the International Waterbird Census (IWC).

**Data set 1.** 

Column label	Column description
id (Event core, Occurrence extension)	Unique identifier for the record; used both for event and occurrence tables to link the data properly.
eventID (Event core, Occurrence extension)	Identifier linking the occurrence to a corresponding sampling event.
eventDate (Event core)	The date or date range during which the event occurred, formatted as ISO 8601 (e.g. "2002-11-10" or "2003-01-10/2003-01-20").
samplingProtocol (Event core)	Method used during sampling. Here described as "ad hoc observation" for expert-based field observations.
samplingEffort (Event core)	Free-text description of the effort used in conducting the event, for example, "5_observer-hours".
sampleSizeValue (Event core)	Numeric value representing the size of the area surveyed – here 35.3.
sampleSizeUnit (Event core)	The unit of measurement for sampleSizeValue – here "square_kilometre".
countryCode (Event core)	Two-letter ISO country code, for example, "PL" for Poland.
country (Event core)	Name of the country in which the event took place – for example, "Poland".
decimalLatitude (Event core)	Latitude of the survey location (in decimal degrees, WGS84).
decimalLongitude (Event core)	Longitude of the survey location (in decimal degrees, WGS84).
geodeticDatum (Event core)	The geodetic datum for the coordinates; usually "WGS84".
coordinateUncertaintyInMetres (Event core)	Horizontal spatial uncertainty in metres for the given coordinates.
basisOfRecord (Occurrence extension)	The nature of the record. Here, all records are "HumanObservation".
occurrenceID (Occurrence extension)	Unique identifier for the occurrence record, typically formed by combining eventID and species name.
organismQuantity (Occurrence extension)	Number of individuals observed; "0" indicates absence.
organismQuantityType (Occurrence extension)	Type of quantity recorded – in this case "individuals".
occurrenceStatus (Occurrence extension)	Indicates whether the taxon was present or absent during the event. Acceptable values: "present", "absent".
scientificName (Occurrence extension)	The full scientific name of the taxon, including authorship and year at first mention, for example, *Anasacuta* (Linnaeus, 1758).
kingdom(Occurrence extension)	Taxonomic kingdom – here always "Animalia".
taxonRank (Occurrence extension)	The taxonomic rank of the scientific name – for example, "species".
eventRemarks (Event core)	Free-text field providing standardised survey status information. For example: "conducted; exact date" or "not conducted"
locality (Event core)	Free-text description of the specific place where the event occurred. In this dataset, typically given as the name of the lake, for example, "Miedwie".

## Additional information

This long-term dataset not only supports wetland conservation and bird population assessments in Central Europe, but also provides a valuable temporal reference for ecological responses to climate change, human pressures and land-use dynamics. It offers strong potential for integration with broader European waterbird monitoring initiatives (Marchowski et al. 2025a).

## Figures and Tables

**Figure 2.**
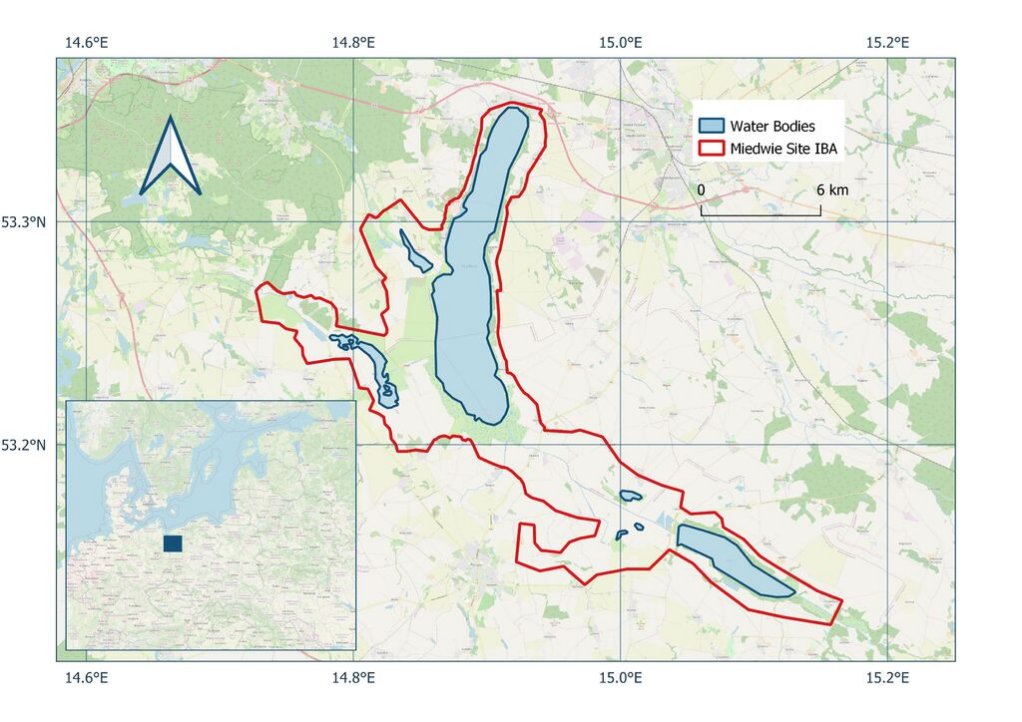
Map of the Miedwie Site Important Bird and Biodiversity Area (IBA) in north-western Poland. The red outline indicates the official IBA boundary, which includes Lake Miedwie and adjacent wetlands and smaller lakes. Blue areas represent major water bodies within the site. The inset shows the site's location within Central Europe.

**Table 1.** Mean population size of each species during the first (2002/2003–2007/2008) and last (2018/2019/2023/2024) six seasons of monitoring, percentage change between these periods and long-term population growth rate (λ) estimated for the period 2002/2003–2024/2025. Trend categories are based on TRIM output: strong increase (↑↑), moderate increase (↑), stable (→), moderate decline (↓), steep decline (↓↓) and uncertain (?). Threat status is provided, based on national and global assessments (CR – Critically Endangered, VU – Vulnerable, NT – Near Threatened, LC – Least Concern, NA – Not Applicable); where different, both are shown. PL – Poland, EU – Europe, GL – global. Population size in each monitoring season was calculated as the average of counts from November, January and March. These seasonal averages were then further averaged across the first six and the last six monitoring seasons to assess long-term changes in abundance.

Species	First6	Last6	% change	λ	Trend	Threat Status
TOTAL	13,888	8,057	-42	0.9758	↓	NA
*Anasacuta* Linnaeus, 1758	16	11	-34	0.9825	↓	CR_PL,_ LC_GL_
*Anascrecca* Linnaeus, 1758	110	22	-80	0.9108	↓	LC
*Anasplatyrchynchos* Linnaeus, 1758	2,128	1,632	-23.3	0.9879	↓	LC
*Anseralbifrons* Scopoli, 1769	3,823	1,626	-57.5	0.9308	↓↓	LC
*Anseranser* Linnaeus, 1758	38	700	1,740	1.1322	↑↑	LC
*Anserfabalis* (Latham, 1787) sensu lato	5,984	2,420	-60	0.9322	↓↓	LC
*Aythyaferina* (Linnaeus, 1758)	152	71	-53.1	0.9585	↓	VU
*Aythyafuligula* (Linnaeus, 1758)	184	304	65	0.9954	→	NT_EU_, LC_GL_
*Aythyamarila* (Linnaeus, 1761)	0	2	1,450	1.1515	?	LC
*Fulicaatra* Linnaeus, 1758	1,324	1,087	-18	0.968	↓	LC
*Marecapenelope* (Linnaeus, 1758)	60	84	41	0.9857	↓	CR_PL,_ LC_GL_
*Marecastrepera* (Linnaeus, 1758)	55	26	-53	1.01	↑	LC
*Mergellusalbellus* (Linnaeus, 1758)	7	10	28	0.9397	↓	LC
*Spatulaclypeata* (Linnaeus, 1758)	4	12	172	1.2025	?	VU_PL_

**Table 2.** Taxa included in the dataset.

Scientific Name	Common Name	Rank	Order	Family
*Anasacuta*	Northern Pintail	species	Anseriformes	Anatidae
*Anascrecca*	Eurasian Teal	species	Anseriformes	Anatidae
*Anasplatyrhynchos*	Mallard	species	Anseriformes	Anatidae
*Marecastrepera*	Gadwall	species	Anseriformes	Anatidae
*Marecapenelope*	Eurasian Wigeon	species	Anseriformes	Anatidae
*Spatulaclypeata*	Northern Shoveler	species	Anseriformes	Anatidae
*Aythyaferina*	Common Pochard	species	Anseriformes	Anatidae
*Aythyafuligula*	Tufted Duck	species	Anseriformes	Anatidae
*Aythyamarila*	Greater Scaup	species	Anseriformes	Anatidae
*Mergellusalbellus*	Smew	species	Anseriformes	Anatidae
*Anserfabalis*	Taiga Bean Goose	species	Anseriformes	Anatidae
*Anserserrirostris*	Tundra Bean Goose	species	Anseriformes	Anatidae
*Anseralbifrons*	Greater White-fronted Goose	species	Anseriformes	Anatidae
*Anseranser*	Greylag Goose	species	Anseriformes	Anatidae
*Fulicaatra*	Eurasian Coot	species	Gruiformes	Rallidae
